# From physical impact to biological information flow: shock wave regulation of extracellular vesicles mediated tissue repair

**DOI:** 10.3389/fcell.2026.1915539

**Published:** 2026-08-18

**Authors:** Shengchao Zhang, Yuling Gao, Jiaqi Zhou, Tong Li, Jingwen Liu, Lan Yang, Qipei Qiao, Xiaoyang Gong, Yong Liu

**Affiliations:** 1 Department of Rehabilitation Medicine, The First Affiliated Hospital of Dalian Medical University, Dalian, China; 2 College of Health-Preservation and Wellness, Dalian Medical University, Dalian, China

**Keywords:** cargo remodeling, extracellular vesicle release, extracellular vesicles, intercellular communication, mechanotransduction, regenerative medicine, shock wave, tissue repair

## Abstract

Shock wave is a tunable pulsed mechanical stimulus that may modulate cellular behavior and tissue microenvironments during repair. However, how localized and transient mechanical inputs are translated into sustained paracrine or remote reparative responses remains incompletely understood. Extracellular vesicles, as mediators of intercellular communication after mechanical stress, may help convert local shock wave-induced cellular responses into transmissible biological signals. This narrative review summarizes current evidence on the shock wave–extracellular vesicle axis, focusing on extracellular vesicle release, cargo remodeling, and repair-associated functions. Available studies suggest that shock wave may promote extracellular vesicle secretion through mechanotransduction-related processes, including the ATP/P2X7R/p38 MAPK axis and nSMase2/ceramide signaling, while also altering microRNA-, protein-, and transcription factor-related cargo. Shock wave-induced or shock wave-preconditioned extracellular vesicles have shown potential pro-angiogenic, anti-apoptotic, antioxidative, immunomodulatory, and matrix-remodeling effects in experimental models of skin wounds, myocardial ischemia, myocardial ischemia-reperfusion, and diabetic bladder dysfunction. Overall, the shock wave–extracellular vesicle axis provides a candidate mechanistic framework for explaining part of shock wave-mediated tissue repair. Further studies should standardize shock wave parameters, improve extracellular vesicle characterization, and establish *in situ* tracing and causal validation.

## 1 Introduction

Shock wave therapy was initially applied for urologic lithotripsy and has been progressively extended in recent years to multiple tissue repair contexts, including orthopedics, sports medicine, rehabilitation medicine, chronic wounds, and ischemic cardiovascular diseases (d’Agostino et al., 2015). Basic and clinical studies have shown that, within an appropriate energy range, shock wave may contribute to tissue repair through processes such as mechanotransduction, angiogenesis, inflammatory regulation, and matrix remodeling (Lv et al., 2023; Kou et al., 2024; Jin et al., 2025). However, as a localized and transient mechanical stimulus, the biological effects of shock wave cannot always be fully explained by the immediate responses of cells within the stimulated region. In particular, the sustained reparative responses, paracrine effects, and benefits in remote tissues observed in some studies suggest that tissue repair after shock wave treatment may involve more complex mechanisms of intercellular communication (Xie et al., 2026).

Extracellular vesicles (EVs) are lipid bilayer-enclosed particles released by cells. They can carry proteins, lipids, mRNAs, miRNAs, and other noncoding RNAs, thereby influencing recipient cell function through paracrine or circulating routes (Kumar et al., 2024). EVs are now recognized as important mediators of intercellular information transfer, and their composition and function are shaped by donor cell type, physiological status, and external stimuli (Dixson et al., 2023). Because EVs participate in multiple tissue repair-related processes, including inflammatory responses, angiogenesis, cell survival, immune regulation, and matrix reconstruction, their roles in regenerative medicine have received considerable attention (Zhang et al., 2024). Meanwhile, mechanical stimulation can affect EVs biogenesis, release, and cargo composition (Thompson and Papoutsakis, 2023), providing a new perspective for understanding the paracrine effects induced by shock wave therapy.

In recent years, several studies have begun to examine the relationship between shock wave and EVs. Existing evidence indicates that shock wave can affect the release quantity or cargo composition of EVs derived from different cell sources. These EVs have shown certain roles in repair-associated processes, including angiogenesis, anti-apoptosis, antioxidative stress, immune regulation, and matrix remodeling, and preliminary validation has been obtained in vitro and *in vivo* models of skin wounds, myocardial ischemia, myocardial ischemia-reperfusion injury, and diabetes-associated organ dysfunction (Xie et al., 2026; Gollmann-Tepeköylü et al., 2020; Yang et al., 2021; Wu et al., 2025; Zheng et al., 2026). These findings suggest that EVs may participate in part of the tissue repair effects induced by shock wave; however, the current evidence remains limited. Differences exist among studies in shock wave parameters, donor cell sources, EVs isolation and characterization methods, sampling time points, and functional evaluation systems. Therefore, whether shock wave regulation of EVs constitutes a stable, reproducible, and broadly applicable repair mechanism requires further validation.

Based on the existing literature, this review focuses on the shock wave–EV axis as an emerging research direction. First, the basic characteristics of shock wave as a mechanical stimulus and its potential links with EV responses are summarized, followed by an overview of the foundational roles of EVs in tissue repair and responses to the mechanical microenvironment. On this basis, this review highlights existing experimental evidence showing that shock wave induces EV release, alters EV cargo composition, and participates in tissue repair. The potential roles of related cargo in angiogenesis, inflammatory regulation, cytoprotection, and matrix reconstruction are analyzed, and representative disease models are used to evaluate research progress and limitations in the evidence base. Finally, key issues facing shock wave-related EV research in therapeutic monitoring, parameter optimization, EV product development, and clinical translation are discussed. Herein, this review aims to clarify the current evidence base, possible mechanisms, and translational boundaries of shock wave-induced EV involvement in tissue repair.

## 2 Methods

### 2.1 Literature screening strategy

Although this review adopted a narrative review framework for evidence synthesis and mechanistic discussion to enable a more in-depth and flexible analysis of biological mechanisms, the retrieval, screening, and reporting of core studies were conducted with reference to the principles of transparency, rigor, and reproducibility advocated by PRISMA 2020 (Preferred Reporting Items for Systematic Reviews and Meta-Analyses) (Page et al., 2021). Specifically, PRISMA 2020 was used to guide the reporting of information sources, search strategies, eligibility criteria, and study selection. As this article is a narrative rather than a systematic review, the guideline was applied primarily to improve the transparency of core evidence identification and screening.

A comprehensive computerized search was performed in the PubMed, Web of Science, Embase, and Scopus electronic databases. The search period extended from database inception to June 10, 2026. To ensure high search sensitivity, the search strategy combined database-specific controlled vocabulary, such as MeSH terms in PubMed and Emtree expanded terms in Embase, with free-text terms in titles and abstracts, and strictly included both singular and plural forms of relevant terms. The search strategy was constructed around three core conceptual pools: (1)extracorporeal shock wave stimulation, including Extracorporeal Shockwave Therapy and High-Energy Shock Waves; (2) extracellular vesicles, including extracellular vesicles, exosomes, and microvesicles; and (3) tissue repair and regenerative outcomes, including wound healing, angiogenesis, and regeneration. In addition, the reference lists of included core articles were manually searched to avoid omission of key studies. The detailed search strategy and Boolean logic are presented in Table 1, with PubMed search syntax used as a representative example.

**TABLE 1:** PubMed database search strategy.

Number	Search strategy
#1	“High-Energy Shock Waves” [MeSH Terms] OR “Extracorporeal Shockwave Therapy” [MeSH Terms]
#2	“shock waves” [Title/Abstract] OR “shockwave” [Title/Abstract] OR “shockwaves” [Title/Abstract] OR “ESWT” [Title/Abstract] OR “ECSWT” [Title/Abstract] OR “extracorporeal shock” [Title/Abstract]
#3	#1 OR #2
#4	“Extracellular Vesicles” [MeSH Terms] OR “Exosomes” [MeSH Terms]
#5	“extracellular vesicle” [Title/Abstract] OR “extracellular vesicles” [Title/Abstract] OR “exosome” [Title/Abstract] OR “exosomes” [Title/Abstract] OR “microvesicle” [Title/Abstract] OR “microvesicles” [Title/Abstract] OR “microparticle” [Title/Abstract] OR “microparticles” [Title/Abstract] OR “small extracellular vesicle” [Title/Abstract] OR “small extracellular vesicles” [Title/Abstract] OR “sEV” [Title/Abstract] OR “sEVs” [Title/Abstract]
#6	#4 OR #5
#7	“Wound Healing” [MeSH Terms] OR “Regeneration” [MeSH Terms] OR “neovascularization, physiologic” [MeSH Terms]
#8	“wound healing” [Title/Abstract] OR “tissue repair” [Title/Abstract] OR “regeneration” [Title/Abstract] OR “angiogenesis” [Title/Abstract] OR “ischemia” [Title/Abstract] OR “revascularization” [Title/Abstract]
#9	#7 OR #8
#10	#3 AND #6 AND #9

The complete Boolean query diagram is presented for PubMed. for embase, Web of Science, and Scopus, the strategy employed database-specific field tags and syntax while retaining the same core concepts.

### 2.2 Inclusion and exclusion criteria

#### 2.2.1 Inclusion criteria

Studies were required to meet all of the following criteria for inclusion study type: (1) original research articles based on *in vitro* cellular models or *in vivo* animal models; (2) intervention: the intervention group explicitly received shock wave treatment; (3) effector mediator: the study explicitly included the isolation and characterization of extracellular vesicles (EVs) or exosomes, and evaluated changes in their release quantity or vesicular cargo; (4) functional outcomes: the study verified defined biological functions of shock wave-induced EVs in promoting tissue repair or angiogenesis, attenuating oxidative stress, or regulating inflammatory responses; and (5) language restriction: the full text was published in English.

#### 2.2.2 Exclusion criteria

Studies meeting any of the following criteria were excluded: (1) publication type: conference abstracts, editorials, expert letters, articles reporting only preliminary data without a complete peer-reviewed full text, and other review articles; (2) inconsistent intervention: studies that applied only simple mechanical stress, such as fluid shear stress or static tensile strain, without intervention using a shock wave generator; (3) inconsistent mechanism: studies that investigated only the direct effects of shock wave on tissue repair without isolating extracellular vesicles as a key intermediate mediator; and (4) Quality limitations: studies with major experimental design flaws or severely inadequate EV isolation and characterization methods that failed to meet the basic identification requirements proposed by the International Society for Extracellular Vesicles (ISEV) under the Minimal Information for Studies of Extracellular Vesicles (MISEV) framework (Welsh et al., 2024). The MISEV framework recommends characterizing isolated particles using complementary evidence, including particle morphology and size distribution, EV-associated protein markers, and markers indicating non-EV components or cellular contamination, rather than identifying EVs solely on the basis of particle size or a single marker.

### 2.3 Study screening process

**FIGURE 1:**
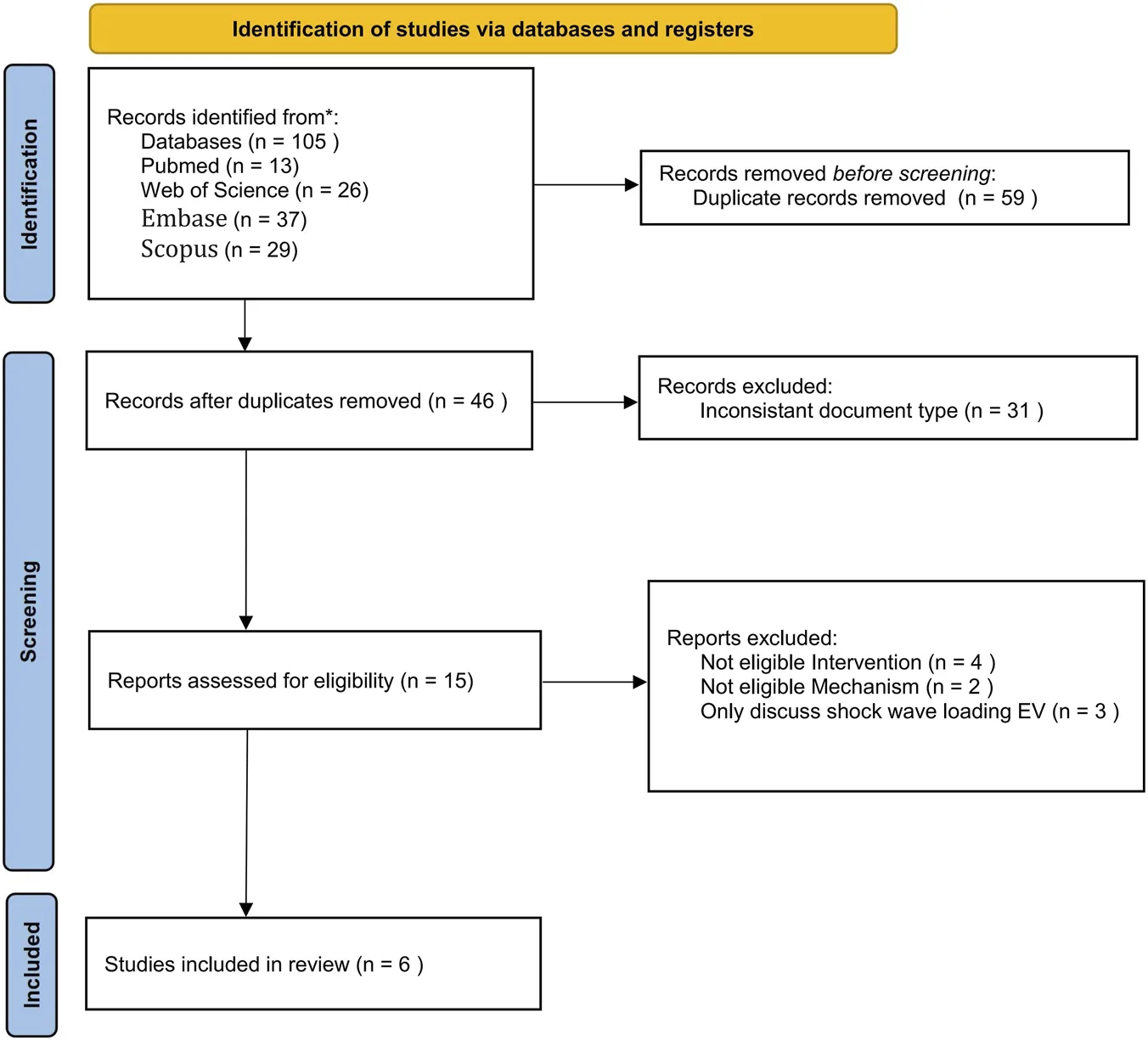
PRISMA 2020 flow diagram for new systematic reviews which included searches of databases and registers only.

The literature screening process was independently conducted in a blinded manner by two researchers. First, all records retrieved from the databases were imported into reference management software (Zotero), and duplicate records were removed through a combination of software-based and manual procedures. Subsequently, the two researchers performed preliminary screening by reviewing article titles and abstracts. For studies that initially met the eligibility criteria, full texts were downloaded and carefully reviewed for final inclusion assessment. During the entire independent blinded screening process, disagreements between the two researchers were resolved through internal discussion. If consensus could not be reached, the study was submitted to a third senior researcher for final arbitration. The detailed screening process is shown in Figure 1.

The original studies ultimately included through the above process constituted the core evidence base for analyzing shock wave regulation of EV release, cargo alterations, and repair-associated functions in this review. Given that a narrative review framework was adopted, in addition to the core included studies, background literature related to EV characterization standards, the physical and acoustic features of shock wave, mechanotransduction pathways, ischemic injury, and tissue repair was appropriately cited in the background and mechanistic discussion sections. This background literature was used primarily to support theoretical interpretation and mechanistic linkage, and does not constitute direct causal evidence that shock wave induces EV-mediated tissue repair. By distinguishing core experimental evidence from mechanistic background evidence, this review seeks to maintain logical completeness while avoiding inferences that extend beyond the scope of existing studies.

## 3 Shock wave: a theoretical interface between mechanical input and EV responses

### 3.1 Overview of shock wave

Shock wave is a type of transient acoustic wave characterized by an extremely short rise time, high peak pressure, and a subsequent negative-pressure phase. Unlike relatively stable mechanical stimuli, such as sustained fluid shear stress, cyclic stretching, or changes in matrix stiffness, shock wave delivers mechanical energy to tissues in a transient, pulsed, and penetrative manner (Auersperg and Trieb, 2020). In tissue repair applications, low-energy shock wave generally does not exert its effects through macroscopic tissue destruction. Instead, it alters the pericellular mechanical microenvironment through microscopic physical effects, including local pressure gradients, acoustic microstreaming, transient cavitation, shear stress, and tensile strain (P et al., 2022; Chen et al., 2022). Shock wave can directly influence cell survival, proliferation, migration, angiogenesis, inflammatory responses, and extracellular matrix remodeling within the treated tissue (d’Agostino et al., 2015). These direct tissue effects are strongly dependent on treatment parameters and tissue context. Within appropriate ranges of energy flux density, pulse number, and treatment frequency, shock wave may induce subinjurious adaptive responses and promote tissue repair (d’Agostino et al., 2015; Wang, 2012). However, when the local mechanical load exceeds the tolerance of cells or tissues, excessive changes in membrane permeability, cavitation, and mechanical stress may lead to cellular injury, microvascular damage, or exacerbated inflammation (Cheng and Wang, 2015). Thus, a clear boundary exists between the therapeutic and injurious energy windows. Shock wave may therefore exert beneficial biostimulatory effects under appropriate conditions but become damaging when unsuitable parameters are applied. These direct responses may occur independently of EVs or act together with soluble paracrine signals and EV-mediated intercellular communication to shape the overall tissue effects of shock wave treatment (Xie et al., 2026). Thereby, shock wave can be understood as an exogenous and parameter-adjustable mechanical perturbation, which converts transient physical energy input into mechanical signals perceptible to cells.

### 3.2 From mechanical stress states to EV regulatory potential

Cells do not merely passively withstand mechanical stimulation; rather, they sense external mechanical changes through alterations in membrane tension, reorganization of membrane microdomains, mechanosensitive ion channels, integrin–focal adhesion complexes, and cytoskeletal networks (Di et al., 2023; Lim and Harraz, 2024). Local mechanical perturbations induced by shock wave may act on the cell membrane and its associated mechanosensory structures. Through mechanosensitive Ca^2+^ signaling, ATP release, cytoskeletal involvement, and activation of pathways such as ERK1/2 and p38 MAPK, exogenous mechanical stimuli may be converted into intracellular biochemical signals (Takahashi et al., 2019; Weihs et al., 2014; Szwarc-Hofbauer et al., 2025). These early events shift stimulated cells from a relatively quiescent state toward a mechanical stress- and repair-associated response state, providing an upstream signaling basis for subsequent biological effects, including angiogenesis, inflammatory regulation, cell survival, and matrix remodeling.

Notably, mechanotransduction affects not only intracellular signaling pathways and functional phenotypes, but may also alter the cellular membrane system, endosomal trafficking, cytoskeletal dynamics, and secretory activity (Di et al., 2023). EV biogenesis and release depend on membrane deformation, mobilization of the endosomal system, vesicle transport, and membrane fusion, all of which are characterized by marked mechanical sensitivity (Jin et al., 2022). Therefore, the mechanical stress state induced by shock wave may not only alter the immediate local responses of stimulated cells, but may also convert transient mechanical input into transmissible intercellular communication signals by regulating EV release and cargo selection (Xie et al., 2026; Gollmann-Tepeköylü et al., 2020). Based on this logic, EVs can be regarded as important candidate mediators linking shock wave mechanical stimulation to tissue repair responses.

## 4 EVs: nanocarriers decoding mechanical force responses

### 4.1 Overview of EVs

EVs are membrane-enclosed particles released by cells and surrounded by a lipid bilayer. They can carry various bioactive molecules, including proteins, lipids, nucleic acids, and metabolites, and participate in intercellular information transfer (Kumar et al., 2024; Welsh et al., 2024). Because the composition and function of EVs are influenced by the donor cell type, physiological state, and external stimuli, their cargo profiles are often regarded as an important reflection of changes in donor cell status. According to the MISEV2023 recommendations of the International Society for Extracellular Vesicles (ISEV), in the absence of definitive evidence for biogenesis, EVs should not be directly defined as exosomes, microvesicles, or other specific subtypes solely on the basis of particle size or commonly used markers. Instead, operational nomenclature should be adopted, such as small EVs or medium/large EVs, or EVs should be described in combination with particle size, density, surface markers, donor cell type, and isolation method (Welsh et al., 2024). Given that the standards for EV isolation, characterization, and nomenclature remain incompletely harmonized in current shock wave-related studies, this review uses EVs as the general term while retaining the terminology used in the original studies where appropriate.

### 4.2 Significance of EVs in tissue repair

Compared with individual soluble factors, EVs are characterized by combinatorial signal transfer. First, EVs carry multiple bioactive molecules simultaneously, thereby exerting multi-target and multi-pathway regulatory effects on recipient cells (Kalluri and LeBleu, 2020). Second, EV surface molecules can participate in recipient cell recognition, adhesion, and uptake, conferring a certain degree of donor cell- and tissue microenvironment-dependent specificity to EV-mediated information transfer (Mathieu et al., 2019). Third, EV cargo composition is influenced by factors such as donor cell type, metabolic status, inflammatory stimulation, hypoxia, and mechanical stress; therefore, EV cargo can partially reflect the injury or repair state of donor cells (Dixson et al., 2023). This feature suggests that EVs may serve not only as effector mediators of reparative responses, but also as candidate biomarkers for evaluating changes in the tissue microenvironment.

At the functional level, EV involvement in tissue repair is mainly reflected in four aspects. First, EVs can regulate angiogenesis (Babaei et al., 2021). EVs derived from mesenchymal stem cells, platelets, or macrophages can influence endothelial cell proliferation, migration, tube formation, and vascular stability, thereby contributing to reperfusion of ischemic tissue and granulation tissue formation in wounds (Bian et al., 2019; Tang et al., 2021; Gangadaran et al., 2020). Second, EVs can regulate inflammatory responses and immune cell phenotypes (Buzas, 2023). Appropriate inflammation during the early phase of tissue injury facilitates the clearance of necrotic tissue and pathogen-associated signals; however, persistent inflammation impairs repair and promotes fibrosis. EVs may participate in inflammatory amplification, inflammatory resolution, or the establishment of immune tolerance by affecting the functions of macrophages, dendritic cells, neutrophils, and lymphocytes (Buzas, 2023). Third, EVs can enhance the survival capacity of injured cells. EVs from multiple sources have exhibited anti-apoptotic, antioxidative stress, and mitochondrial homeostasis-maintaining effects in models associated with hypoxia, oxidative stress, or ischemia-reperfusion (Bister et al., 2020). These effects are particularly important for the post-injury survival of cardiomyocytes, endothelial cells, neurons, and skin cells. Fourth, EVs can participate in extracellular matrix remodeling. EVs may influence fibroblast activation, collagen deposition, matrix degradation, and scar formation, thereby affecting the structural quality of repaired tissue and long-term functional recovery (An et al., 2021; Ding et al., 2023).

### 4.3 Sensitivity of EVs to the mechanical microenvironment

Early EV research often linked vesicle release to fundamental cellular processes, including reticulocyte maturation, clearance of membrane proteins, endosomal trafficking, and exocytosis of multivesicular bodies (MVBs) (Pan and Johnstone, 1983). However, emerging evidence from mechanobiology indicates that EV biogenesis mechanisms, including ESCRT complex-mediated sorting, Rab GTPase-dependent trafficking, and SNARE complex-mediated membrane fusion, exhibit high mechanosensitivity to the mechanical microenvironment in which cells reside (Wu et al., 2023; Huang et al., 2022; Carvalho Ferraz et al., 2025). For example, physiological fluid shear stress or cyclic tensile strain can not only increase EV secretion from endothelial cells or fibroblasts by several-fold, but also specifically alter the enrichment patterns of miRNA transcripts, thereby enhancing pro-angiogenic and immunomodulatory functions (Li et al., 2025). These mechanical stimulation models are more suitable for dissecting the fundamental mechanisms of EV biogenesis in specific tissue microenvironments. By contrast, shock wave is more closely aligned with physical therapeutic approaches already used in the field of tissue repair.

### 4.4 Comparison between shock wave-induced EVs and other EV induction strategies

Various biological and engineering stimuli can alter the release quantity, subpopulation composition, and cargo profiles of EVs (Dixson et al., 2023; Thompson and Papoutsakis, 2023). Positioning shock wave-induced EVs within these regulatory approaches helps clarify that the key scientific question is not whether shock wave is superior to other induction methods, but whether shock wave, as a clinically implementable pulsed mechanical stimulus, can participate in tissue repair-related intercellular communication through EV release and cargo remodeling. Therefore, it is necessary to first compare the input characteristics, major EV alterations, and translational boundaries of different EV regulatory strategies, so as to define the rational positioning of the shock wave–EV axis, as shown in Table 2.

**TABLE 2:** Input signal characteristics, major action interfaces, EV-related changes, and key limitations of different EV induction/modulation strategies.

Induction/modulation strategy	Input signal characteristics	Major action interfaces	Main EV-related changes	Scientific questions that can be addressed	Major limitations
Shock wave	Short-duration, pulsed stimulus with a high pressure gradient and tissue penetration capacity	1. Membrane tension 2. Mechanosensitive Ca^2+^ signaling 3. ATP/P2X7R 4. p38 MAPK 5. Endosomal/multivesicular body mobilization	1. EV release kinetics 2. Remodeling of pro-reparative cargo	How local mechanical therapy is converted into transmissible paracrine reparative signals	High parameter heterogeneity
Fluid shear stress	Continuous or cyclic hydrodynamic stimulation	1. Endothelial mechanosensing 2. Cytoskeleton3.KLF/YAP/TAZ-related pathways	1. Endothelial-derived EVs 2. miRNAs associated with vascular homeostasis	How the blood-flow microenvironment shapes EV-mediated communication	Limited clinical operability
Cyclic stretch/compression	Cyclic tissue deformation	1. Integrins 2. Cytoskeleton 3. Matrix–cell connections	1. ECM remodeling 2. Inflammation-related cargo 3. Repair-related cargo	How mechanical loading affects EV profiles	Differences in time scales and mechanical spectra
Hypoxia/oxidative stress	Metabolic or injury-related stimulation	1. HIF 2. ROS 3. Inflammatory pathways	1. Anti-apoptosis-related cargo 2. Angiogenesis-related cargo 3. Inflammation-related cargo	How the injury microenvironment induces adaptive or pathological EV changes	Unclear safety boundaries
Inflammatory factor/cytokine pretreatment	Biochemical stimulation	1. NF-κB 2. STAT 3. Immunoregulatory networks	Immunomodulatory cargo	How the immune microenvironment shapes EV function	Relatively distant from mechanisms of physical therapy
Hypoxia/three-dimensional culture/biomaterial-based culture	Microenvironmental engineering	1. Reprogramming of cellular state 2. Matrix sensing	1. Enhanced EV yield 2. Enhanced regenerative function of EVs	How to prepare functionally enhanced EVs	More closely related to EVs product manufacturing
Electroporation/ultrasound/direct transfection	Applied to isolated EVs or donor cells	1. Membrane permeabilization 2. Introduction of exogenous cargo	1. Exogenous nucleic acids 2. Drug loading	How to improve the drug delivery capacity of EVs	Mainly focused on engineered loading

Compared with sustained shear stress or cyclic tensile strain, shock wave differs in its input time scale and mechanical spectrum. It is therefore more suitable for discussing EV responses after pulsed mechanical perturbation, rather than serving as a replacement for classical mechanobiology models (Thompson and Papoutsakis, 2023; Zeng et al., 2024). Compared with hypoxia, inflammatory factors, or pharmacological preconditioning, shock wave better preserves the mechanistic attributes of physical therapy; however, EV alterations induced by shock wave still need to be distinguished from cellular injury, stress-associated particle release, and increases in non-EV components (Song et al., 2025; Yuan et al., 2025; Chen et al., 2025). Compared with engineering strategies such as electroporation, ultrasound-assisted loading, or direct transfection, shock wave-induced EVs emphasize endogenous EV biogenesis and cargo selection after donor cells are exposed to mechanical stimulation, whereas shock wave-assisted EV loading represents a distinct category of formulation engineering (Du et al., 2023; Kim et al., 2024). Therefore, the purpose of comparing different EV regulatory approaches is not to establish a simple hierarchy of superiority, but to clarify the evidence boundaries and translational positioning of the shock wave–EV axis. This axis may serve as a mechanistic framework for explaining part of the tissue repair effects of shock wave therapy, and may also provide a candidate tool for EV-based therapeutic monitoring, parameter optimization, and the preparation of functionally enhanced EV products. However, before standardization is achieved across disease models, shock wave parameters, donor cell sources, and EV isolation methods, shock wave-induced EVs should still be regarded as a mechanistic hypothesis requiring validation, rather than an established therapeutic mechanism.

Furthermore, a more fundamental consideration should not be overlooked: therapeutic shock waves are not the only form of pulsed acoustic exposure encountered by humans. Individuals may also be involuntarily exposed to non-therapeutic, high-intensity pulsed acoustic sources, including explosions, blasting operations, and sonic booms. In military personnel repeatedly exposed to low-level blast waves over prolonged periods, the levels of the pro-inflammatory mediators TNF-α and IL-6 were significantly elevated in brain-derived EVs, whereas the anti-inflammatory mediator IL-10 was reduced. Moreover, these EV-associated inflammatory markers were correlated with neurobehavioral symptoms and structural brain alterations (Stone et al., 2024; Edwards et al., 2022). Such findings raise the possibility that harmful exposures may also engage mechanosensitive EV-related signaling pathways and thereby contribute to tissue injury. From the opposite perspective, these observations highlight the importance of defining a safe therapeutic window for shock wave treatment to prevent a shift from regenerative stimulation toward tissue damage.

## 5 Candidate biological mechanisms linking shock wave-remodeled EVs to tissue repair

On the basis of the theoretical framework described above, shock wave-induced EV promotion of tissue repair may involve two interrelated levels. First, shock wave may promote EV release from donor cells, thereby enhancing paracrine signal output. Second, shock wave may remodel EV cargo, resulting in the enrichment of molecules associated with angiogenesis, cytoprotection, immune regulation, and matrix remodeling.

### 5.1 A multistage model of shock wave-induced EV release

**FIGURE 2:**
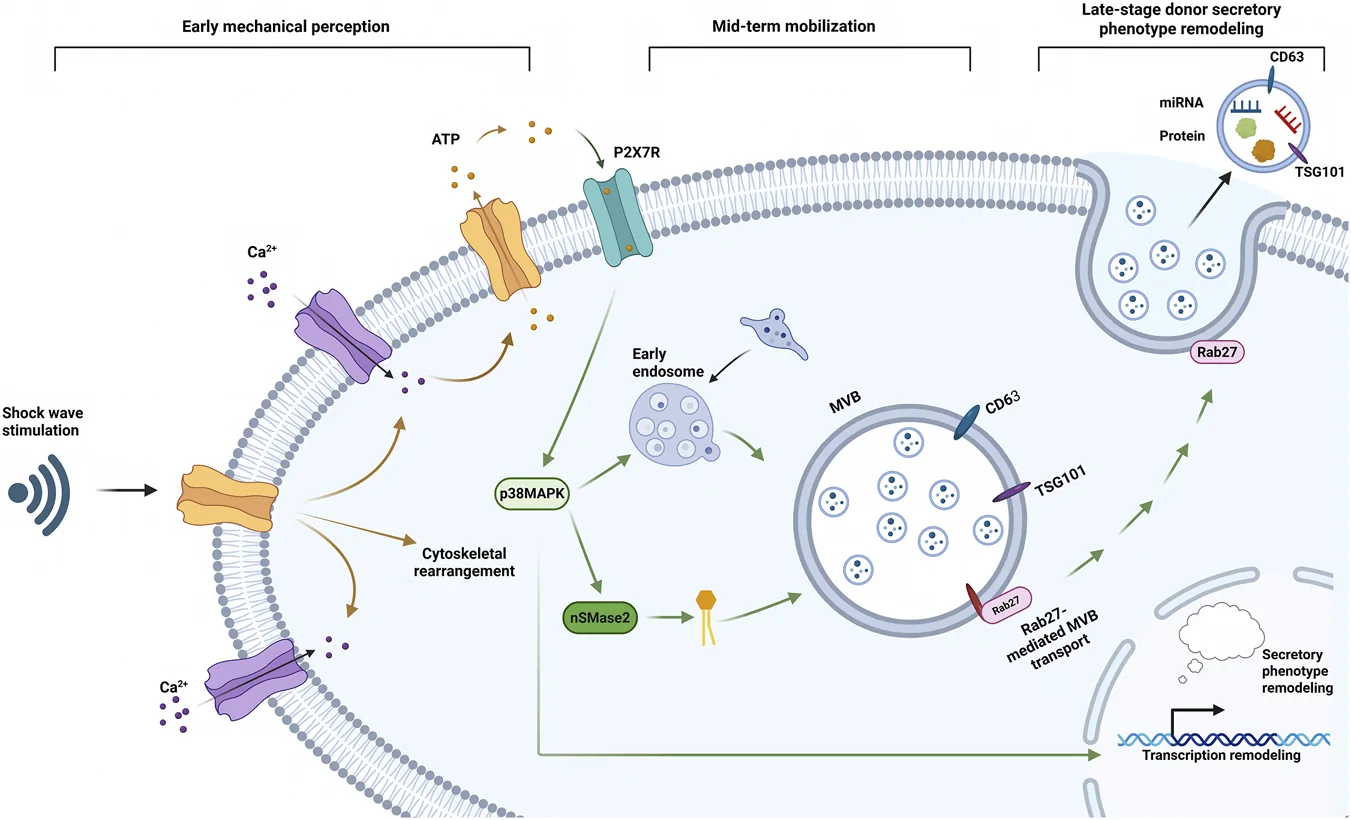
Working model of shock wave-associated EV release. Early mechanosensing phase. Shock wave stimulation acts on the plasma membrane and may activate mechanosensitive ion channels, Ca^2+^ influx, cytoskeletal remodeling, and extracellular ATP release. ATP may then signal through autocrine/paracrine activation of P2X7R and downstream p38 MAPK, forming a potential early ATP/P2X7R/p38 MAPK axis related to vesicle secretion. Intermediate mobilization phase. Early mechanosensory signals may promote endosomal system mobilization, typically within 1–4 h after stimulation. Activation of p38 MAPK and nSMase2 may facilitate early endosome-to-MVB maturation and intraluminal vesicle formation. Rab27-associated trafficking may then support MVB transport and fusion with the plasma membrane, leading to rapid EV release enriched in markers such as CD63 and TSG101. Late remodeling of the donor-cell secretory phenotype. Acute mechanical triggering and intermediate secretory execution may be followed by transcriptional and secretory remodeling of donor cells, extending vesicular responses into later time windows, such as 24 h, and potentially affecting selective loading of cargos such as miRNAs and proteins. Note: This diagram represents a conceptual working model. Current evidence is insufficient to establish the depicted signaling nodes as a single, strictly linear causal pathway.

Mechanical stimulation can promote EV biogenesis and release by regulating cell membrane tension, cytoskeletal remodeling, membrane receptor activation, intracellular Ca^2+^ influx, and related processes (Thompson and Papoutsakis, 2023; Ae et al., 2018). Different forms of mechanical signals, including fluid shear stress, cyclic stretching, compressive loading, and ultrasound stimulation, have been shown to affect multivesicular body formation, membrane fusion, and vesicle secretion, thereby altering the release quantity and biological functions of EVs (Thompson and Papoutsakis, 2023; Li et al., 2024). As a mechanical stimulus characterized by transient high pressure, rapid energy transfer, and tissue penetration capacity, shock wave may induce EV release and alter paracrine signal output by regulating mechanotransduction in donor cells. Based on currently fragmented evidence, shock wave-induced EV release is conceptualized in this review as a multistage mechanobiological process involving early mechanical sensing, intermediate endosomal/multivesicular body mobilization, and late remodeling of the donor-cell secretory phenotype, as shown in Figure 2. It should be emphasized that this model is primarily derived from integrated inference across different cell types and experimental systems, and has not yet been fully validated within a single system. Therefore, when discussing increased EV release, it is necessary to distinguish genuine enhancement of vesicle biogenesis and secretion from mechanical stress, membrane injury, apoptotic body release, or increased non-vesicular particles.

During the early response phase, shock wave may theoretically initiate vesicle release-related signaling through events such as altered membrane tension, activation of mechanosensitive ion channels, Ca^2+^ influx, cytoskeletal rearrangement, and extracellular ATP release. Among these processes, the ATP/P2X7R/p38MAPK axis currently has relatively substantial direct functional evidence in studies of shock wave-induced EV release. Xie et al. (Xie et al., 2026) showed that shock wave stimulation of bone marrow mesenchymal stem cells (BMSCs) increased ATP levels in the cell supernatant and activated P2X7R/p38MAPK signaling. ATP hydrolase, a P2X7R inhibitor, and a p38MAPK inhibitor all attenuated the shock wave-induced increase in small EV release, suggesting that ATP-mediated purinergic signaling may represent an important early step by which shock wave initiates vesicle secretion.

During the intermediate response phase, shock wave may further promote mobilization of the endosomal system, formation of multivesicular bodies (MVBs), generation of intraluminal vesicles (ILVs), and fusion of MVBs with the plasma membrane, thereby enhancing the secretion of endosome-derived EVs. Gollmann-Tepeköylü et al. (Gollmann-Tepeköylü et al., 2020) showed that, after shock wave treatment, the number of EVs in the culture medium of human umbilical vein endothelial cells (HUVECs) did not increase nonspecifically immediately after exposure, but was significantly increased at 1 h and remained elevated at 4 h. Coronary artery endothelial cells (CAECs) derived from aged donors exhibited a similar rapid release response at 4 h. Further analysis showed that shock wave treatment increased the number of MVBs in HUVECs and was accompanied by upregulation of nSMase2 expression, suggesting that the nSMase2/ceramide pathway may participate in ILV formation within MVBs and their subsequent extracellular release. In bone marrow mesenchymal stem cells (BMSCs), EV particle concentration was increased after shock wave stimulation and was accompanied by upregulation of CD63, TSG101, and Rab27 expression (Xie et al., 2026). Because CD63 and TSG101 are associated with the biogenesis of endosome-derived EVs (van Niel et al., 2011; Colombo et al., 2013), whereas Rab27 is involved in the regulation of vesicle secretion (Ostrowski et al., 2010), these findings suggest that shock wave may promote mobilization of the EV secretory system. In particular, the above changes at 4 h indicate that shock wave can enhance EV generation and release at the organelle level. It should be noted that these molecular and morphological findings support the involvement of the endosomal/MVB pathway in EV release induced by shock wave, but in the absence of direct biogenesis-tracing evidence, they are still insufficient to prove that all released vesicles are exosomes in a strict biogenetic sense.

The response in the same direction observed in CAECs further suggests that this mechanism is not specific to HUVECs, but may be preserved in coronary artery endothelial cells that more closely approximate the clinical context of myocardial ischemia. The release kinetics within the 1–4 h time window suggest that the increase in EVs induced by shock wave does not primarily reflect instantaneous membrane rupture or cellular debris release. Instead, it more likely represents a secretory execution phase in which early mechanosensing signals are further converted, through processes such as Ca^2+^ signaling, ATP/purinergic signaling, and cytoskeletal rearrangement, into endosomal system mobilization, MVB formation, and MVB–plasma membrane fusion. In these studies, the observed peak in EV release within 1–4 h suggests a rapid secretory response upon acoustic stimulation, potentially distinct from immediate membrane rupture or long-term transcriptional remodeling.

During the late response phase, the vesicular response induced by shock wave may progress from acute mechanical triggering and intermediate secretory execution to sustained vesicle release over several hours to 24 h. The core issue at this stage is not to revisit mechanosensing or MVB mobilization, but to emphasize the temporal extension of the release process. *In vitro*, vesicle-like structures were still observed in human fascial fibroblasts 24 h after shock wave treatment (Pirri et al., 2022). This finding suggests that the vesicular response induced by shock wave is not limited to immediate release after stimulation, but may extend into a later time window. However, evidence for the late phase should be interpreted cautiously. The structures observed in that study were vesicle-like structures enriched in hyaluronic acid and type I and III collagen, and were described mainly on the basis of morphology, histochemistry, immunostaining, and TEM imaging (Pirri et al., 2022). Thus, these findings cannot directly demonstrate the persistence of *de novo* vesicle release at 24 h.

In summary, shock wave-induced EV release is better understood as a dynamic process jointly involving mechanosensing, endosomal/multivesicular body mobilization, and remodeling of the donor-cell secretory phenotype. In addition, Pölzl et al. established a standardized water bath protocol for EV isolation after shock wave treatment (Pölzl et al., 2020). This methodological study suggests that detection of shock wave-induced EV release is highly dependent on mechanical coupling, treatment parameters, and sampling time control. Therefore, subsequent studies comparing EV release quantity and function should rigorously report *in vitro* shock wave exposure conditions and isolation workflows. However, existing studies do not yet demonstrate that the signals described above constitute a unified and linear causal pathway; therefore, this model should be defined only as a working model.

### 5.2 Molecular profiles and reparative functional orientation of shock wave-remodeled EV cargo

**FIGURE 3:**
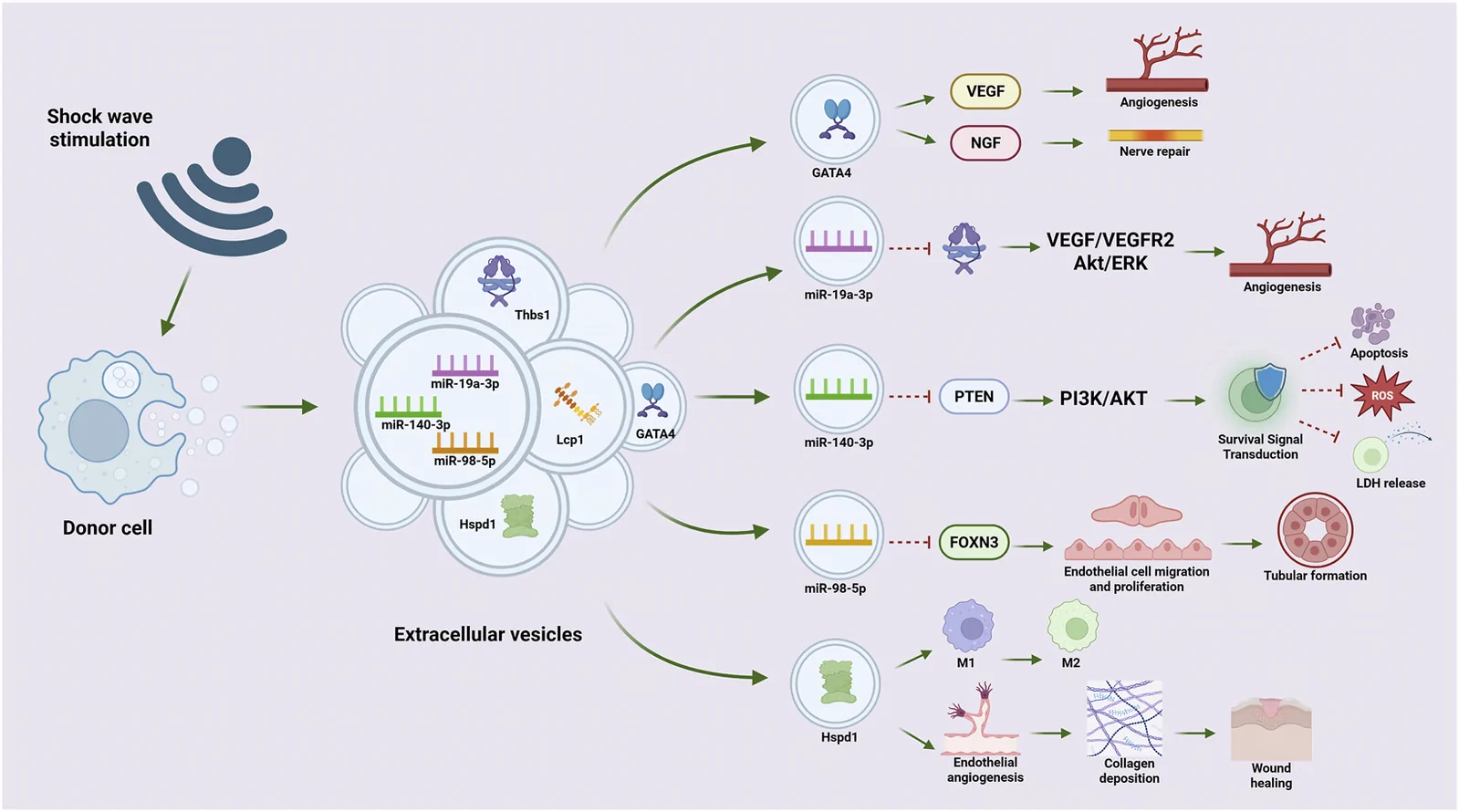
Cargo remodeling and potential reparative orientation of shock wave-associated EVs. Shock wave stimulation may remodel EV miRNA, protein, and transcription-related cargo, thereby influencing repair-associated responses in recipient cells. EV-enriched miR-19a-3p has been reported to target TSP-1/Thbs1 and is associated with enhanced VEGF/VEGFR2–Akt/ERK-related angiogenic signaling. miR-140-3p may target PTEN and activate PI3K/AKT signaling, contributing to cell survival and reduced apoptosis, oxidative stress, and injury-related responses. miR-98-5p may target FOXN3 and promote endothelial migration, proliferation, and tube-like structure formation. In addition, shock wave-induced EVs may show altered protein cargo, including increased Lcp1, Thbs1, and Hspd1. Thbs1-enriched EVs have been linked to macrophage M1-to-M2 polarization, endothelial angiogenesis, collagen deposition, and wound healing. Shock wave-preconditioned adipose-derived mesenchymal stromal cells may also activate the donor-cell FAK/p38 MAPK–GATA4 axis, accompanied by increased GATA4, VEGF, and NGF, suggesting potential paracrine roles in angiogenesis and neural repair. Overall, the reparative effects of shock wave-associated EVs may involve coordinated cargo networks related to angiogenesis, cytoprotection, immunomodulation, matrix remodeling, and tissue healing. Some mechanisms remain based mainly on *in vitro* models or candidate cargo validation and require further *in vivo* confirmation.

The ability of shock wave-induced EVs to promote tissue repair may be related not only to an increase in the number of EVs released by cells, but also to the contents delivered within these vesicles. The available evidence is summarized in Figure 3.

#### 5.2.1 Key miRNA axes regulated by shock wave in EVs

##### 5.2.1.1 miR-19a-3p/TSP-1 axis

miR-19a-3p is an endogenous small noncoding RNA that belongs to the miR-19 family and is also a member of the canonical miR-17–92 gene cluster (Mogilyansky and Rigoutsos, 2013). Functionally, miR-19a-3p can act as a network-type regulatory factor. Based on classical studies of the miR-17–92 cluster, miR-19a/miR-19b represents a functionally important branch of this cluster and is frequently involved in the regulation of cell proliferation, apoptosis, differentiation, inflammatory responses, angiogenesis, and tissue remodeling. Its common downstream targets and related pathways include PTEN/PI3K-AKT, BCL2L11, TGF-β-related signaling, and certain inflammatory transcriptional networks, enabling this miRNA to influence cell survival, stress responses, and the tissue microenvironment (Mogilyansky and Rigoutsos, 2013). In the study by Gollmann-Tepeköylü et al. (Gollmann-Tepeköylü et al., 2020), miRNA profiling identified 31 miRNAs that were enriched in EVs released from shock wave-treated endothelial cells. Subsequent quantitative PCR validation confirmed the enrichment of miR-7i, miR-19a, and miR-19b, whereas the enrichment of miR-301b was not validated. Among the candidate miRNAs subjected to functional evaluation, miR-7i and miR-19b did not show detectable pro-angiogenic activity, whereas miR-19a-3p enhanced endothelial tube formation and proliferation.

Additional gain- and loss-of-function experiments further supported the involvement of miR-19a-3p under these experimental conditions. Antisense oligonucleotide-mediated inhibition of miR-19a-3p suppressed shock wave-associated endothelial tube formation and proliferation *in vitro* and reduced angiogenic responses in a mouse hindlimb ischemia model. Transfection of endothelial cells with miR-19a-3p resulted in decreased TSP-1 expression at both mRNA and protein levels, accompanied by increased VEGFR2 expression. Meanwhile, EVs released from shock wave-treated endothelial cells activated the pro-angiogenic Akt and ERK signaling pathways, leading to enhanced endothelial cell proliferation and tubular structure formation (Gollmann-Tepeköylü et al., 2020). These observations are consistent with enhanced VEGF/VEGFR2-related signaling and downstream Akt/ERK activation during angiogenesis (Shaw et al., 2024).

Human studies provide additional evidence supporting the disease relevance of miR-19a-3p, although its role as a mediator of shock wave therapy in patients has not yet been established. Circulating miR-19a has been reported to be altered in patients with acute myocardial infarction and has been investigated as a potential diagnostic biomarker (Zhong et al., 2014). Elevated serum miR-19a-3p levels have also been associated with the presence and severity of asymptomatic carotid artery stenosis, as well as subsequent cerebrovascular ischemic events (Liu et al., 2021). In a dietary intervention study involving overweight or obese participants, longitudinal changes in circulating miR-19a-3p were associated with changes in estimated atherosclerotic cardiovascular disease risk (Xue et al., 2024). These observations support the clinical relevance of miR-19a-3p as a biomarker associated with vascular and cardiovascular diseases.

Notably, the biological effects of EV-associated miR-19a-3p appear to be context-dependent. In contrast to the pro-angiogenic effects observed with EVs released from shock wave-treated endothelial cells, another experimental study (Gou et al., 2020) reported that miR-19a-3p in cardiomyocyte-derived EVs inhibited endothelial proliferation and angiogenesis by targeting HIF-1α, whereas inhibition of miR-19a-3p improved angiogenesis and cardiac function after myocardial infarction. These seemingly conflicting findings may reflect differences in EV-producing cell types, injury conditions, recipient cells, miRNA abundance, and downstream targets. Therefore, current evidence supports the involvement of miR-19a-3p in EV-mediated vascular regulation; however, a universally beneficial miR-19a-3p/TSP-1 mechanism across different tissues and experimental contexts has not yet been established.

##### 5.2.1.2 miR-140-3p/PTEN/PI3K/AKT axis

MiR-140-3p is a mature microRNA derived from the 3′arm of precursor miR-140 and belongs to the class of small noncoding RNAs that exert their effects through post-transcriptional regulation of target gene expression (Wu et al., 2019). Yang et al. (Yang et al., 2021) compared EVs derived from shock wave-treated and untreated endothelial colony-forming cells and identified 18 differentially expressed miRNAs with relatively high abundance, including 8 upregulated and 10 downregulated miRNAs. miR-140-3p was selected for further validation because it was highly abundant and significantly upregulated in both shock wave-treated donor cells and their EVs, while its predicted target genes were associated with the significantly enriched PI3K/AKT signaling network. Accordingly, this review focuses primarily on miR-140-3p based on the magnitude of its differential expression, its abundance within EVs, its pathway relevance, and the completeness of subsequent functional validation; this emphasis does not imply that the other differentially expressed miRNAs lack potential biological roles. Further experiments showed that EVs derived from shock wave-treated endothelial colony-forming cells were taken up by H9c2 cardiomyoblast-like cells, resulting in increased intracellular miR-140-3p levels, reduced PTEN expression, and enhanced phosphorylation of PI3K and AKT. A dual-luciferase reporter assay confirmed the direct targeting of the 3′untranslated region of PTEN mRNA by miR-140-3p. Overexpression of miR-140-3p partially reproduced the effects of these EVs in enhancing cell viability, inhibiting apoptosis, reducing oxidative stress, and attenuating NF-κB-related inflammatory responses, whereas inhibition of miR-140-3p weakened these effects (Yang et al., 2021). These findings support the involvement of miR-140-3p in EV-associated cytoprotection within this experimental system.

Human studies further suggest that miR-140-3p has potential clinical relevance to cardiovascular disease. Elevated miR-140-3p levels have been detected in the plasma and peripheral blood mononuclear cells of patients with early acute coronary syndrome (Li et al., 2017). In a cohort of patients with coronary artery disease, circulating miR-140-3p was associated with the risk of cardiovascular mortality and prognostic discrimination in patients with acute coronary syndrome (Karakas et al., 2017). Alterations in miR-140-3p levels within plasma EVs from patients with stable coronary artery disease have also been reported to be associated with disease severity and vascular endothelial barrier function (Han et al., 2024). However, these studies reflect clinical associations between miR-140-3p and cardiovascular disease status or prognosis and do not establish its involvement in EV-mediated effects following shock wave therapy in patients.

##### 5.2.1.3 miR-98-5p/FOXN3 axis

miR-98-5p is a let-7 family-related microRNA that regulates gene expression at the post-transcriptional level by binding to the 3′UTR of target gene mRNAs (Hazari et al., 2024). Wu et al. (Wu et al., 2025) performed miRNA sequencing of EVs derived from oxygen-glucose deprivation/reoxygenation-treated H9c2 cardiomyoblast-like cells and found that shock wave treatment induced differential expression of 39 miRNAs, including 20 upregulated and 19 downregulated miRNAs. miR-98-5p showed the highest abundance in EVs from the shock wave-treated group, and its increase was further validated by qRT-PCR; it was therefore selected for subsequent functional and target-gene analyses. The study examined the effects of miR-98-5p on both donor cardiomyoblast-like cells and recipient endothelial cells. Inhibition of miR-98-5p attenuated the protective effects of EVs derived from shock wave-treated cells on H9c2 cells subjected to oxygen-glucose deprivation/reoxygenation and reduced their ability to promote HUVEC proliferation, migration, invasion, and tube formation. In endothelial cells, miR-98-5p directly bound to the 3′untranslated region of FOXN3 mRNA and downregulated FOXN3 expression. When miR-98-5p was inhibited, FOXN3 silencing partially restored the associated angiogenic phenotypes (Wu et al., 2025). Thus, the direct evidence for the miR-98-5p/FOXN3 axis primarily supports the regulation of endothelial cells by cardiomyoblast-derived EVs.

Clinical studies suggest that miR-98-5p is associated with myocardial ischemia and coronary microvascular injury, although the direction of change has not been consistent across studies. In patients with ST-segment elevation myocardial infarction undergoing primary percutaneous coronary intervention, higher serum miR-98-5p levels were associated with impaired microvascular reperfusion (Hu et al., 2021). Other studies have reported reduced miR-98-5p levels in patients with acute myocardial infarction and have linked this miRNA to the regulation of cardiomyocyte apoptosis, inflammation, and oxidative stress (Ma et al., 2021; Neiburga et al., 2021). These discrepancies suggest that the clinical significance of miR-98-5p may depend on disease stage, sample type, and cellular source. Current human evidence supports the relevance of miR-98-5p as a molecule associated with ischemic cardiovascular injury, but a clinical link between miR-98-5p and the response to shock wave therapy has not yet been established.

#### 5.2.2 Shock wave-remodeled EV protein cargo participates in tissue repair

Shock wave can also remodel the protein cargo of EVs. Xie et al. (Xie et al., 2026) performed DIA-based proteomic analysis of EVs derived from the tibial osteotomy group and small EVs derived from the group receiving shock wave stimulation at the tibial osteotomy site, and found that the EV protein profile was markedly altered after shock wave stimulation. Enrichment analysis suggested that the differentially expressed proteins were mainly involved in cellular processes, binding/catalytic activity, and ribosome- and translation-related pathways, indicating that shock wave not only increased EV release but may also alter EV protein composition and potential biological effects. Further screening and validation showed that Lcp1, Thbs1, and Hspd1 are currently best regarded as validated upregulated candidate proteins, whereas their causal roles in the pro-reparative effects of shock wave-induced EVs require further clarification.

Among these candidate proteins, Thbs1 currently has the most substantial functional evidence as an effector molecule. Xie et al. (Xie et al., 2026) constructed Thbs1-enriched engineered small EVs and found that Thbs1-enriched small EVs enhanced IL-4-induced M2 macrophage polarization and promoted human umbilical vein endothelial cell proliferation, migration, and tube formation. *In vivo* experiments further showed that Thbs1-enriched small EVs accelerated wound closure, increased epidermal regeneration, collagen deposition, and CD31-positive neovascularization, and regulated the transition of wound macrophages from an M1 phenotype toward an M2 phenotype (Xie et al., 2026).

It should be noted that TSP-1/Thbs1 is a multidomain matricellular protein that participates in angiogenesis, inflammatory responses, and extracellular matrix remodeling through multiple receptors, including CD36, CD47, and integrins, as well as through the regulation of TGF-β activation, cell–matrix adhesion, and protease activity (Lawler and Lawler, 2012; Sweetwyne and Murphy-Ullrich, 2012; Kyriakides and Maclauchlan, 2009). Accordingly, its net effect on tissue repair is highly dependent on cell type, temporal stage, and the local microenvironment. Classical studies have generally regarded TSP-1 as an endogenous inhibitor of angiogenesis that suppresses endothelial cell proliferation and survival through CD36-related signaling (Dawson et al., 1997). However, TSP-1 also contributes to apoptotic cell clearance, resolution of inflammation, epithelial cell migration, and tissue remodeling and may therefore facilitate repair during specific stages of tissue injury (Savill et al., 1992; Wilson et al., 2024). miR-19a-3p-mediated suppression of TSP-1 occurs primarily in the context of endothelial cell angiogenesis (Gollmann-Tepeköylü et al., 2020), whereas Thbs1-enriched EVs may promote wound repair mainly through macrophage polarization, matrix remodeling, and stage-dependent vascular regulation (Xie et al., 2026). Therefore, TSP-1 should not be simply defined as either a pro-angiogenic or anti-angiogenic molecule.

#### 5.2.3 Donor-cell transcriptional regulatory axes and EV functional remodeling

In addition to miRNA and conventional protein cargo, shock wave may also influence the molecular composition and biological function of EVs by regulating key mechanotransduction signaling axes in donor cells. Zheng et al. (Zheng et al., 2026) reported that shock wave preconditioning of adipose-derived mesenchymal stromal cells (ADSCs) activated the FAK/p38 MAPK-GATA4 axis and further upregulated the expression level of the transcription factor GATA4, thereby promoting increased secretion of VEGF and NGF. Meanwhile, increased GATA4 expression was also detected in shock wave-induced EVs, suggesting functional remodeling of EV cargo composition. This phenomenon suggests that shock wave may regulate the transcriptional program of donor cells through the FAK–p38 MAPK–GATA4 mechanotransduction axis and further influence the loading of repair-associated molecules in ADSCs, thereby enhancing their paracrine reparative potential. Unlike classical miRNA-mediated post-transcriptional regulatory mechanisms, GATA4-related evidence suggests that the functional enhancement of shock wave-induced EVs may partially depend on the upregulation of key transcription factors and the coordinated activation of downstream vascular and neural repair factors. Because GATA4 has been shown to participate in regulating the expression of angiogenesis-related genes such as VEGF, its enrichment in EVs may provide an important paracrine explanation for the effects of shock wave in promoting neovascularization and neural repair (Zheng et al., 2026).

## 6 Evidence for the shock wave–EV axis in disease models

At present, studies on the involvement of shock wave-induced EVs in tissue repair remain limited, and existing evidence is mainly derived from a small number of animal models and *in vitro* cellular models. Differences also exist among these models, and the relevant studies are summarized in Table 3. Therefore, this section reviews the potential roles and research limitations of shock wave-induced EVs in tissue repair based on representative models.

**TABLE 3:** Summary of key experimental evidence for the involvement of shock wave-induced EVs in tissue repair.

Study	EVs yield	EVs source	Model type	Shock wave parameters	Key cargo/pathways	Key findings
Gollmann-Tepeköylü et al., 2020	Increase (high variance)	Endothelial cells	MouseLAD ligation-induced myocardial ischemia model	——	miR-19a-3p/TSP-1; Akt/ERK	Angiogenesis↑; myocardial fibrosis ↓; Left ventricular function ↑
Yang et al., 2021	No change	ECFCs	H9c2cell hypoxia/reoxygenation injury model	0.09 mJ/mm^2^, 500 shots	miR-140-3p/PTEN/PI3K/AKT	Oxidative stress ↓ H/R injury ↓
Wu et al., 2025	No increase	OGD/R-H9c2	H9c2 cell OGD/R injury model	0.12 mJ/mm^2^, 500 shots	miR-98-5p/FOXN3	Angiogenesis ↑ OGD/R injury ↓
Xie et al., 2026	Significant increase	Plasma/BMSCs	Rat dorsal foot full-thickness skin wound model	3 bar, 300 impulses 5 Hz	Thbs1; Lcp1; Hspd1 ATP/P2X7R/p38MAPK	Woundhealing↑; angiogenesis ↑; M2 polarization ↑
Zheng et al., 2026	Not measured	ADSCs	rat model of diabetic bladder dysfunction	0.1 mJ/mm^2^, 800 impulses	FAK/p38MAPK/GATA4	Angiogenesis ↑; nerve repair ↑; bladder function improved ↑

### 6.1 Skin wounds: model evidence for circulating EV-Mediated remote repair

The skin wound model provides relatively representative *in vivo* evidence for the shock wave–EV axis. Xie et al. (Xie et al., 2026) established a model involving proximal tibial ring osteotomy with shock wave stimulation (TOE), with tibial osteotomy alone without shock wave treatment serving as the corresponding control. Unlike direct treatment of the wound, a key feature of this model was that mechanical stimulation was applied to bone tissue, whereas the healing endpoint was set at a distal dorsal foot skin wound. This design was used to test whether mechanical stimulation of bone tissue could affect ectopic skin repair through circulating mediators. The results showed that TOE treatment improved not only ipsilateral wound healing but also repair of the contralateral dorsal foot wound, suggesting that this effect was not merely local but more likely involved reparative signals capable of circulating transmission.

Multiple complementary experimental findings support the involvement of circulating EVs in this remote reparative process. TOE treatment increased the abundance of circulating EVs. Pharmacological inhibition of EV release attenuated the wound-healing effects of TOE. Isolated TOE-associated EVs preferentially accumulated in the wound area *in vivo*, and supplementation with these EVs also promoted wound repair. Collectively, these findings provide converging evidence that circulating EVs participate in and partially mediate TOE-associated remote tissue repair.

### 6.2 Myocardial ischemia: evidence for myocardial repair by shock wave-preconditioned endothelial cell-derived EVs

The myocardial ischemia model provides *in vivo* rescue evidence for shock wave-preconditioned EVs, rather than direct validation of endogenous EVs induced by *in situ* cardiac shock wave. Gollmann-Tepeköylü et al. (Gollmann-Tepeköylü et al., 2020) showed that shock wave promoted the release of EVs with pro-angiogenic activity from endothelial cells. Injection of these EVs into the peri-infarct region after LAD ligation in mice improved preservation of left ventricular function, attenuated post-infarction fibrosis, and enhanced angiogenesis-related phenotypes.

The key contribution of this study lies in demonstrating that shock wave stimulation can confer ischemic myocardial repair activity on endothelial cell-derived EVs, thereby providing *in vivo* evidence in the context of myocardial ischemia for shock wave-preconditioned EVs as a cell-free therapeutic strategy.

### 6.3 Myocardial ischemia-reperfusion: *in vitro* protective evidence for shock wave-preconditioned EVs

Current studies related to myocardial ischemia-reperfusion remain mainly at the level of *in vitro* cellular models. Existing evidence indicates that, regardless of whether donor cells are cardiomyocyte-like cells after hypoxia/reoxygenation injury or endothelial progenitor cell subpopulations with angiogenic potential, shock wave preconditioning can enhance the biological activity of their derived EVs, resulting in anti-injury or pro-vascularization effects in recipient cells (Yang et al., 2021; Wu et al., 2025).

The value of these studies lies in extending the shock wave–EV axis from a single pro-angiogenic phenomenon to the context of ischemia-reperfusion-related cytoprotection, suggesting that shock wave may coordinate cardiomyocyte survival, endothelial responses, and reconstruction of a reparative microenvironment by remodeling the functional states of EVs from different cellular sources.

### 6.4 Diabetic bladder dysfunction: model evidence for shock wave-preconditioned EVs combined with biomaterial-based targeted delivery

The pathological alterations of diabetic bladder dysfunction (DBD) are mainly characterized by concurrent degeneration of bladder microvasculature and peripheral nerve injury (Wittig et al., 2019). As described above, shock wave preconditioning promoted the enrichment of the transcription factor GATA4 in adipose-derived mesenchymal stromal cell-derived EVs and synchronously upregulated VEGF and NGF expression in endothelial cells and neural cells. In *ex vivo* and *in vitro* experiments, administration of these EVs increased vascular endothelial tube formation and significantly extended neurite length in cultured major pelvic ganglion (MPG), confirming their dual pro-vascular and neuroreparative capacity (Zheng et al., 2026).

For the bladder, a dynamic hollow organ, conventional simple intravesical instillation of EVs can be readily washed out by periodic urine filling and voiding, resulting in a short effective retention time (GuhaSarkar and Banerjee, 2010). To achieve effective local delivery, the study (Zheng et al., 2026) used a thermosensitive magnetic chitosan nanoparticles (CSNP) hydrogel as an EV delivery vehicle. This material remained liquid at room temperature to facilitate instillation, transformed into a gel state at body temperature, and contained magnetic nanoparticles. In a DBD rat model induced by streptozotocin combined with a high-fat diet, the investigators transurethrally instilled CSNP hydrogel encapsulating shock wave-induced EVs and applied an external magnetic field *in vivo*. This approach successfully anchored the hydrogel to the bladder wall, enabled sustained EV release, and resisted urinary washout. In the *in vivo* outcome assessment after intervention, conscious cystometry showed that CSNP-targeted EV delivery significantly improved the abnormally prolonged voiding interval in DBD rats and restored maximal voiding pressure. Histological immunofluorescence results showed restoration of capillary density within the detrusor and a significant increase in the number of S100-positive nerve fibers in the bladder submucosa in the intervention group. This disease model not only provides direct functional evidence for shock wave-preconditioned EVs in repairing the neuro–smooth muscle axis, but also indicates that, in the clinical translation of visceral organs, physically reprogrammed EVs usually need to be combined with biomaterials possessing specific physical properties, such as thermosensitivity and magnetic targeting, to overcome organ barriers and achieve an effective local concentration.

## 7 Translational perspectives and challenges

Although existing studies have begun to outline a theoretical framework in which the shock wave–EV axis participates in tissue repair and microenvironmental remodeling, a substantial evidentiary gap remains between proof-of-concept findings and clinical translation. It should first be recognized that the evidence provided by different disease models is not at the same level and does not address the same translational paradigm. Studies of cutaneous wounds have primarily investigated whether circulating EVs participate in ectopic wound repair following shock wave stimulation of a distant tissue (Xie et al., 2026). Myocardial ischemia studies have evaluated the reparative potential of EVs derived from endothelial cells preconditioned with shock wave *in vitro* and subsequently administered *in vivo* (Gollmann-Tepeköylü et al., 2020). Studies related to myocardial ischemia/reperfusion have mainly used *in vitro* cellular systems to examine shock wave-induced remodeling of EV cargo and the resulting cytoprotective or pro-angiogenic effects (Yang et al., 2021; Wu et al., 2025). In diabetic bladder dysfunction, shock wave-preconditioned EVs have further been combined with hydrogels, magnetic targeting, and local sustained-release systems (Zheng et al., 2026). Thus, current studies support distinct scientific propositions at different levels, including the involvement of EVs in remote repair, enhancement of EV function by shock wave preconditioning, and biomaterial-assisted EV delivery. However, these findings cannot yet be integrated into a continuously validated causal chain linking *in situ* shock wave treatment, endogenous EV release, target-tissue uptake, and tissue repair.

The limitations of the existing disease models further define the translational boundaries of this evidence. In the TOE-associated cutaneous wound model, EV-release inhibition, *in vivo* accumulation, and EV supplementation experiments collectively support the participation and partial mediating role of circulating EVs in remote repair. However, GW4869 is not a completely specific inhibitor of EV release, and plasma EVs are characterized by complex cellular origins and substantial subpopulation heterogeneity. Therefore, the relative contribution of EVs compared with soluble factors and other circulating mediators remains unclear (Xie et al., 2026). In addition, an acute full-thickness skin wound model cannot fully reproduce the impaired perfusion, neuropathy, persistent inflammation, and infectious burden characteristic of diabetic foot ulcers, infected wounds, or ischemic chronic wounds.

In the myocardial ischemia model, intramyocardial administration of EVs derived from HUVECs preconditioned with shock wave *in vitro* demonstrated the therapeutic potential of functionally enhanced EVs. However, this experimental paradigm essentially represents an exogenous, functionally enhanced EV therapeutic strategy and does not directly demonstrate that clinical cardiac shock wave therapy mediates myocardial repair by inducing endogenous EV release *in vivo* (Gollmann-Tepeköylü et al., 2020). Current myocardial ischemia/reperfusion studies are also based predominantly on *in vitro* systems involving H9c2 cells, HUVECs, and ECFCs and therefore cannot fully reproduce coronary perfusion, immune-cell recruitment, neurohumoral regulation, metabolic remodeling, or multicellular spatial interactions *in vivo* (Yang et al., 2021; Wu et al., 2025; Lindsey et al., 2021).

Similarly, although the diabetic bladder dysfunction model further combined shock wave-preconditioned EVs with thermosensitive hydrogels, magnetic targeting, and local sustained-release delivery systems and demonstrated improvements in bladder function and tissue repair, it evaluated the therapeutic potential of shock wave-preconditioned EVs delivered in combination with biomaterial-based systems rather than an endogenous EV-mediated repair process following *in situ* bladder shock wave stimulation (Zheng et al., 2026). Thus, this model supports the feasibility of combining enhanced EV preparations with delivery platforms but cannot be simply extrapolated as complete validation of an *in situ* shock wave–EV axis. Future translational studies should therefore validate these distinct experimental paradigms separately rather than directly equating the therapeutic efficacy of enhanced EV preparations with the mediating role of endogenous EVs during *in situ* shock wave treatment.

Based on the current evidence system, clinical translation in this field may follow three potential paths. The corresponding translational barriers and Frontier directions are summarized in Table 4:

1. Dynamic features of EVs in local body fluids or the circulation may be used as liquid biopsy biomarkers for evaluating the therapeutic efficacy of shock wave and monitoring prognosis.
2. Noninvasive shock wave parameters may be optimized to stimulate the cascade release of endogenous functional EVs in target organs *in situ*.
3. Donor cells may be preconditioned with shock wave *in vitro* to prepare physically reprogrammed and functionally enhanced EV products for intervention as advanced therapeutic products.

**TABLE 4:** Core barriers and emerging solutions in clinical translation.

Clinical translation pathway	Core translational barriers	Emerging solutions
Liquid biopsy biomarkers (monitoring therapeutic efficacy and prognosis)	1. High background noise in complex body fluids 2. Difficulties in identifying EV tissue-of-origin specificity 3. Lack of validation in large-scale prospective cohorts	1. Introduction of single-vesicle high-throughput sequencing 2. Application of AI and machine learning for multi-target screening
*In situ* induction of endogenous EV release (tissue microenvironment remodeling)	1. Acoustic energy attenuation in deep tissues *in vivo* 2. Risk of off-target effects in surrounding healthy tissues 3. Difficulty in standardizing optimal stimulation parameters and frequencies	1. Development of high-precision focused acoustic devices 2. Integration of high-resolution *in vivo* imaging guidance 3. Construction of personalized computational dosimetry models
Enhanced EV biopharmaceuticals (advanced therapeutic products)	1. Rapid loss in dynamic *in vivo* environments (short half-life of naked EVs) 2. Lack of scalable GMP-compliant purification technologies 3. Cellular heterogeneity leading to batch-to-batch potency variability	1. Combination with biomaterials such as hydrogels or magnetic targeting systems for controlled release 2. Establishment of potency evaluation systems aligned with FDA/EMA standards 3. Expansion to human organoids and non-human primate validation

Compared with the first two paths, the translational threshold for shock wave-preconditioned EV products is undoubtedly the highest. Although *in vitro* studies have demonstrated that shock wave can confer stronger anti-apoptotic and pro-angiogenic activities on EVs (Yang et al., 2021; Wu et al., 2025), this approach has moved beyond the scope of conventional physical medicine and into the field of biopharmaceutical development. Its clinical advancement must systematically address pharmaceutical issues, including donor-cell heterogeneity, scalable vesicle isolation and purification, potency assays, batch-to-batch consistency, and long-term toxicology, under stringent regulatory frameworks, such as FDA/EMA guidance for cell-derived products (Gimona et al., 2017).

It should be particularly emphasized that, as revealed by translational challenges in complex models such as DBD, simple delivery of naked EVs in highly dynamic *in vivo* microenvironments, such as hollow organs subjected to continuous urinary washout, blood vessels exposed to high shear stress, and myocardium undergoing continuous contraction and relaxation, often results in reduced therapeutic efficacy because of an extremely short half-life and low retention in the target region. Therefore, clinical translation of in vitro-preconditioned EVs will inevitably require deep integration with advanced biomaterials, such as thermosensitive hydrogels and magnetic nanocarriers. Coupling shock wave-mediated physical and mechanical reprogramming of cells with material engineering of delivery vehicles to overcome spatial physical barriers in specific tissues and achieve spatiotemporally controlled EV release in lesion regions represents an essential route toward clinical translation in this direction. At present, related evidence is largely limited to *in vitro* models and small animals. In the future, highly consistent validation of safety and efficacy in human-derived organoids and nonhuman primate models will be required before progression to Good Manufacturing Practice (GMP)-compliant formulation development.

To accelerate this process, future studies urgently need to address the following core barriers.

First, standardized systems for shock wave intervention and multidimensional EV characterization should be established by leveraging multi-omics technologies and artificial intelligence (AI) algorithms, thereby addressing the field-wide challenge that published data are difficult to compare across studies.

Second, the cross-scale transduction mechanisms connecting mechanical forces with biochemical signals should be dissected, and molecular maps of mechanosensitive channel-mediated directional sorting of EV cargo should be precisely delineated. For example, spatial mechanotranscriptomic studies have begun to jointly analyze local intercellular tension, intracellular pressure, and transcriptional programs at single-cell resolution, providing a methodological basis for mapping the spatial relationships between tissue mechanical states and biochemical responses (Hallou et al., 2025). Meanwhile, cross-tissue single-cell transcriptomic analyses have been used to systematically characterize the expression patterns of genes associated with EV biogenesis, secretion, and cargo regulation across different cell types (LaRocca and Lark, 2025). More recent studies further suggest that mechanosensitive channels such as PIEZO1 may participate in the regulation of small EV release and may also influence EV molecular composition and biological functions (Annalisa et al., 2026).

Third, rigorous pharmacokinetic studies should be conducted, and high-resolution *in vivo* imaging technologies should be used to track, in real time, the targeted distribution and metabolic trajectories of EVs and their combined delivery systems *in vivo*.

Fourth, tissue-specific clinical indications should be precisely matched. For ischemic heart disease, the focus should be placed on pro-vascular network remodeling and anti-fibrotic effects. For chronic nonhealing wounds, both immune polarization and matrix remodeling should be considered. For complex neurogenic, smooth muscle, and microvascular degenerative lesions associated with conditions such as diabetic complications, emphasis should be placed on evaluating the bidirectional and synergistic rescue networks through which delivery materials and enhanced EV complexes support neural axonal regeneration and target tissue morphogenesis.

## 8 Conclusion

Overall, shock wave-induced EVs are more appropriately regarded as a regenerative medicine framework in which mechanical stimulation regulates intercellular communication, rather than as an established therapeutic modality. Current evidence suggests that shock wave may participate in angiogenesis, inflammatory regulation, cytoprotection, and tissue repair by modulating EV release, cargo composition, and functional states, thereby providing a new mechanistic perspective for understanding the biological effects of shock wave therapy. Evidence from different disease models further indicates that shock wave-induced EVs may serve not only as a means of regulating endogenous reparative signaling but also as a basis for the development and delivery of functionally enhanced EV preparations. However, the available evidence is still derived predominantly from *in vitro* studies, animal models, and proof-of-concept experiments, and the relationships among shock wave parameters, EV alterations, underlying mechanisms, and tissue repair outcomes remain to be further clarified. Future studies should strengthen the standardization of experimental parameters and EV characterization frameworks to improve comparability across studies and should further evaluate the sustained efficacy, safety, and clinical relevance of shock wave-induced EVs through systematic *in vitro* and *in vivo* investigations. In parallel, elucidating how mechanical stimulation regulates EV biogenesis and selective cargo loading will help deepen our understanding of the molecular basis of shock wave–EV-mediated tissue repair and advance this emerging field.
